# Women in Industry

**Published:** 1920-11-20

**Authors:** 


THE PUBLIC WELL-BEING—(*continued*).

### Women in Industry.

### HAVE THEY COME TO STAY?

A widely discussed public health problem, greatly magnified by the war and not solved by the stoppage of hostilities, is that of women in industry. Though complete national statistics of women employed in industry are not available, an investigator in behalf of the *International Journal of Public Health* has been able to make a rough survey which discloses marked differences in the number of women workers and the conditions under which they work. In Japan (1) about two-thirds of the factory workers, or about half a million, are women and girls. The U.S. census for 1910 (2) gave the general figure of 8,000,000 women engaged in "gainful occupations," while the number of men in the same class was about 30,000,000. In 1914 (2) 16.7 per cent. of those employed in manufacturing industries were women. During the war the number of women workers greatly increased, though no complete statistical inquiry was made. The increase was even greater in France, Italy, and Great Britain. In Great Britain (3) the increase in the number of women workers over pre-war figures has been estimated as nearly 50 per cent., taking all occupations into account.

#### France's Post-War Problems.

Of the countries engaged in war each one has had its own post-war attitude towards women in industry. Public opinion in many countries has decreed that returning soldiers should be given preference in employment, but in open competition, and with the inducement of wages sufficient to permit of a higher standard of living, it has not been proved that women will readily step aside from the positions which experience has shown them they can fill. France's dangerously low birth-rate and decreased population cause her to view the problem from a different angle. The national attitude seems to be to discourage women in industry. The excess of the female population over the male population in France at the present time is approximately two millions, which would seem to indicate that those women must pursue a livelihood and that a large percentage will be found in industry.

#### Suitable and Unsuitable Work.

Dr. Janet Campbell has expressed the view that women war workers stood the work as well as they did owing to good wages enabling them to feed and clothe themselves well, to healthy factory conditions, and to welfare and health supervision. If similar demands had been made upon women working under pre-war factory conditions they could not have been met to the same extent, if at all, without causing an immensely greater amount of fatigue and permanent injury to health. "The whole experience (of the war) tends to show that light sedentary work is not by any means always the most suitable for women, that operations involving a change of posture are preferable, and that, given adequate nutrition, many women would have better health and greater physical vigour if they followed more active occupations."

Measures of prevention against ill-effects from the employment of women are divided into restriction of hours of employment and provisions governing the conditions of work. "In most countries there is a certain class of people who denounce legislation in regard to the employment of women, and advocate unrestricted competition for men and women." A more general opinion seems to be that women, as yet an unorganised class of workers, need legislative protection to prevent unscrupulous exploitation. A modern standard of working hours for women sought by health workers and social reformers alike is a forty-eight-hour week, and in some cases a forty-four-hour week, with a Saturday half-holiday. In our view, there is every probability that in the near future this model standard will be raised still higher for women workers as a class.

